# Engaging the New Frontier

**DOI:** 10.3389/fpsyg.2021.527301

**Published:** 2021-04-20

**Authors:** Darren C. Treadway

**Affiliations:** Daemen College, Amherst, NY, United States

**Keywords:** editor and scholar behavior, organizational psychology, organizational behavior, leadership, gig economy, virtual work environments, artificial intelligence

Five years ago, the *Organizational Psychology Special Section* of *Frontiers in Psychology* was launched by founding Editor Richard Boyatzis. At that time, he challenged contributors to focus on the “how” and “what” of Organizational Psychology, and to “go where no one has gone before.” He envisioned the discipline as both boundary spanning and boundary breaking. Answering his challenge, authors have published in excess of 800 articles in the journal since its inception.

The quality and efficiency of the *Frontiers* review process and the promise of the open access concept has led to the journal's impressive global reach, impact factors, and overall readership. Focusing on the goals of access and impact, The *Organizational Psychology Section* is now poised to provide the platform through which the most universal and pressing issues can be rigorously addressed and effectively delivered to those most affected by these challenges.

Unlike the “hard sciences,” the individuals and collectivities organizational scientists study are constantly changing and adapting to their environments. Thus, the accurate prediction of human or group behavior is an imperfect pursuit from which multiple theoretical lens may offer divergent, yet equally valid, predictions. Despite this realization, all too often, established paradigms, understandings, and assumptions are accepted without appropriate concern for context or alternative explanation. This has been perhaps never more evident than with the rapidity of change in organizations around the world we currently are witnessing.

As we move into the third decade of the twenty-first century, the world offers many new challenges for organizations and those of us who study them. Broadly, we might be wise to investigate these new forms of organization and employment, and determine how they impact, and are impacted by, the changing expectations of the modern employee. With that in mind, I offer some challenges, the further study of which may benefit both science and practice. This is not meant to be an exhaustive list, but offers a glimpse into the new frontiers that organizational psychologists could more fully address in the next 5 years. I offer the following for your consideration:

- Artificial Intelligence (AI): AI is transforming the workplace in subtle but highly impactful ways. Human Resource functions such as hiring, onboarding, and training are being recreated by AI innovations. AI is also aiding productivity by reducing repetitive task demands and reducing overall efficiency of data utilization in decision-making. With that being said, organizations need to better understand how employees interact with, react to, and utilize this technology. This research stream has implications for the new models of decision-making, team dynamics, and/or leadership.
- Virtuality: At 1 time, virtual work meant simply doing your current job … at home. As technology has progressed, so has the nature and richness of such virtual work. Technological enhancement has helped organizations overcome some of the relational issues initially associated with such employment relationships, and these improvements account for a continuing increase in the prevalence of such work. Indeed, 75% of professionals worldwide are involved in remote work at least 1 day a week (International Workplace Group, 2019). What might this mean for not only how we design work processes, but how we envision organizations, and the nature and meaning of work itself?
- Gig work: What seems like not too long ago, employees signed on with an employer after their schooling ended, and spent their careers within the same organization. However, decades of layoffs and downsizings, generational differences in motivation, and the technological advances noted above have fundamentally altered the nature of the employment relationship. Individuals engaging in short-term, skill-based projects for one or more employers (gig work) currently comprise 20–30% of all workers in the United States and the European Union (McKinsey Global Institute, 2016). These participation numbers are only expected to grow in the coming decades as employers use new staffing models to reduce expenses, and workers increasingly desire more control over their work-life balance. With identity and commitment as core concepts in organizational psychology, it will be interesting to see how this “maker” culture impacts individuals' understanding of their identity and the underlying psychological contracts related to employment.
- Social Change: The access to information and knowledge has driven modern social change movements from climate change to #MeToo. There are advocacy groups for nearly every cause, which are constantly fighting for limited resources and attention. Although we know, anecdotally, a great deal about the most successful of these movements, we know much less about the “whys” of involvement and the “hows” of success. If information gives us the power to change the world, it is incumbent upon organizational scholars to make sure that there are research-based models to assist in translating individual enthusiasm into substantive change. Topics such as leadership, networks, and culture might be on the forefront of these new understandings of social progress.
- Diversity: Technology and information access are shrinking the world. In many ways, this has become a means for increased understanding and empathy across cultural boundaries. However, we cannot ignore the role that technology and social networks play in promoting and mobilizing hate and prejudice. Moving forward, a more balanced understanding of the interplay among diversity, identity, technology, and culture appears warranted.
- Work-Life Balance: Much has been made about potential generational differences in motivation related to Millennials. Yet, with current employees expected to retire at a later age, we know very little about engaged older workers. These potentially competing value sets demand a deeper understanding of innovative organizational designs, work roles, and idiosyncratic relationships to meet the shifting salience of work—life issues across the increasing generational range in organizations.
- Leadership: Leadership scholars continue to generate new theories and models that explain the complexity of effective leader-member relations. The focus of much of this research has been on examining new styles of leadership that reflect an individual leader's inherent morality (e.g., servant leaders, authentic leaders, etc.). Yet, the changing context of work discussed above implies a degree of behavioral flexibility not necessarily included in each leader's style. Indeed, it might be suggested that the shifting and often conflicting demands of today's workplace requires even moral dexterity as well. We necessarily need a more comprehensive understanding of the leader's fluidity in moving between different contexts and coalitions.

These 7 contexts are but a few of the most pressing issues facing organizations today. We have become even more aware of their impact during the COVID-19 pandemic. The *Organizational Psychology* Section demonstrated its promise during this pandemic by launching the first Special Issue related to the impact of the pandemic on the work environment. Whereas, recognition is warranted for our section quickly placing knowledge in the hands of decision-makers, this reactionary model is unsustainable and suboptimum for realizing the potential of organizational psychology.

In a world confronted with crises of health, equity, and morality, it is critical for the field of Organizational Psychology to meet these challenges with forward thinking research. That is, our Grand Challenge is to lead: through our scholarship, our compassion, and our humility. The flood of information brought about by enhanced access to technology, has led, at best, to an overwhelming volume of information that too often rewards the style rather than the substance of a scientific discovery. At its worst, this deluge of data has led to less precise conclusions based on pseudo-scientific information and the suppression of comprehensive scientific findings. Perhaps now more than any time in modern history, organizations, their leaders, and their participants need rigorous science to illuminate a path forward through an increasingly ambiguous environment. It is upon us as organizational scientists to engage these new challenges and offer rigorous research that improves the human condition.

## Author Contributions

The author confirms being the sole contributor of this work and has approved it for publication.

## Conflict of Interest

The author declares that the research was conducted in the absence of any commercial or financial relationships that could be construed as a potential conflict of interest.
